# Assessment of the phytochemical profile and antioxidant activities of eight kiwi berry (*Actinidia arguta* (Siebold & Zuccarini) Miquel) varieties in China

**DOI:** 10.1002/fsn3.2525

**Published:** 2021-08-20

**Authors:** Jiyue Zhang, Jinlong Tian, Ningxuan Gao, Er Sheng Gong, Guang Xin, Changjiang Liu, Xu Si, Xiyun Sun, Bin Li

**Affiliations:** ^1^ College of Food Science Key Laboratory of Healthy Food Nutrition and Innovative Manufacturing of Liaoning Province National R&D Professional Center for Berry Processing Shenyang Agricultural University Shenyang China

**Keywords:** antioxidant, kiwi berry, phenolic compounds, phytochemical

## Abstract

The kiwi berry (*Actinidia arguta*) is a new product on the market that expanding worldwide acceptance and consumption. This widespread interest has created an increasing demand to identify the nutritional and health benefits of kiwi berry. Many studies are being actively conducted to investigate the composition and health‐promoting effects of kiwi berry. In this study, the phytochemical content of free and bound fractions of eight kiwi berry varieties were systematically investigated in order to better understand the potential of this superfood crop. Nine phenolic monomers were identified and quantified by ultrahigh‐performance liquid chromatography coupled with quadrupole time‐of‐flight mass spectrometry and ultrahigh‐performance liquid chromatography‐PAD. Antioxidant activity was further determined via peroxyl radical scavenging capacity and cellular antioxidant activity assays. The free extracts had higher phytochemical contents and antioxidant activities than the corresponding bound extracts among the eight kiwi berry varieties. Bivariate Pearson's and multivariate correlation analyses showed that antioxidant activities were most related to the total phenolic, flavonoid, vitamin C, and phenolic acids contents. The results provide a theoretical basis for the selection of kiwi berry varieties and the utilization of functional foods.

## Graphical abstract

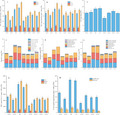

## 1 INTRODUCTION

The genus *Actinidia* (*Actinidiaceae*) is consisted of >50 species. Kiwi berry (*Actinidia arguta*) belongs to the genus *Actinidia* and is a plant that native to southeast Asia, but its cultivars can grow in different regions with harsh climates, soon after became wild fruits extremely appreciated in Europe (Latocha, 2015). Moreover, the kiwi berry become worldwide appreciated mainly because of its high phenolic (Aneta Wojdyło, 2019), vitamin C (Latocha, 2015), carotenoid, and mineral (K, Ca, P, Fe, and Zn) contents (Nishiyama et al., 2005). At present, the global annual production of kiwi berry is about 1600 tons, mainly distributed in the United States, Chile, China, Australia, and European countries such as France, Belgium, the Netherlands, Austria, Switzerland, Germany, and Poland (Latocha, 2017). It is worth noting that there has been amazing growth only in China, which has recently established more than 1,200 hectares of new plantations (Latocha, 2015). There are different varieties of kiwi berry on the market, but the most well‐known are “Geneva,” “Ananasnaya,” “Issai,” “Weiki,” “Jumbo,” “Ken's Red,” and “Maki” (Pinto et al., 2020).

Kiwi berry is very aromatic and has a sweet and intense flavor compared to pineapple blackcurrant, banana, ripe strawberry, and other tropical flavors. It can be used for fresh consumption and/or jam and wine (Kim et al., 2009). Moreover, kiwi berry is much richer in bioactive compounds than many other fruit species and shows stronger antioxidant activities (Wojdyło et al., 2017). In addition, unlike the common kiwifruit (*Actinidia deliciosa*), the edible skin of the kiwi berry allows the whole fruit to be consumed, which increases its nutritional value (Latocha, 2015).

Many studies have shown that there is a positive correlation between the phenolic content and antioxidant activity observed in various fruits (Aneta Wojdyło, 2019; Jiao et al., 2018). In the kiwi berry, phenolic compounds and ascorbic acid greatly enhance its antioxidant properties (Latocha, 2015), and these properties are attributed to the presence of various types of phenolic compounds (Latocha, 2015). Phenolic compounds are small molecules characterized by at least one phenol group in their structure (Limwachiranon et al., 2018). These compounds can be divided mainly into phenolic acids and flavonoids (Ree, 2001). A previous study showed that phenolic acids have a potent antioxidant capacity and anti‐inflammatory effect (Heim et al., 2012); additionally, flavonoids have a high antioxidant activity and are correlated with a reduced risk of chronic diseases (Okarter et al., 2010).

In China, kiwi berry varieties are only known and consumed by locals in certain provinces; therefore, kiwi berry is considered to be an underutilized fruit. Kiwi berry varieties in these study have been ignored by researchers, market systems, and food processing companies. They have not been widely marketed or fully processed and utilized to produce products. Moreover, little research has been done on the phenolic content and the antioxidant activity of kiwi berry (Decorte, 2015; Pinto et al., 2020).

In this study, the total phenolic content (TPC), total flavonoid content (TFC), vitamin C content, and antioxidant activities of eight common kiwi berry varieties in China were determined. In addition, qualitative analysis of phenolics via ultrahigh‐performance liquid chromatography coupled with quadrupole time‐of‐flight mass spectrometry (UPLC‐Q‐TOF‐MS) and quantitative analysis of phenolics via high‐performance liquid chromatography coupled with photodiode array mass spectrometry (HPLC‐PDA‐MS) were performed to determine the phenolic compounds in the kiwi berry samples. Bivariate Pearson's and multivariate correlation analyses were used to establish the correlation between the phytochemical profile and antioxidant activities of eight kiwi berry varieties. The results of this study provide baseline data on the phytochemical profile and antioxidant activity of these kiwi berry varieties in China.

## 2 MATERIALS AND METHODS

### 2.1 Chemicals and reagents

Ascorbic acid, gallic acid, and Folin–Ciocalteu reagent, sodim borohydride (NaBH_4_), aluminum chloride, chlorogenic acid, cyanidin‐3‐*O*‐glucoside, and 2,7‐dichlorodihydrofluorescein diacetate were obtained from Aladdin Industrial Corporation (Shanghai). 2,2′‐Azobis (2‐amidinopropane) dihydrochloride (ABAP) was purchased from Macklin Biochemical Co., Ltd, and 2,7‐dichlorodihydrofluorescein diacetate (DCFH‐DA) was obtained from Sigma Chemical Co. HPLC grade methanol was obtained from Aladdin Co., Ltd. All reagents used were employed for chemical analysis.

### 2.2 Kiwi berry sample preparation

Kiwi berries generally germinate in mid‐April, enter the peak growth period from late May to mid‐June, enter the full bloom period in late June, and mature around August–October. Therefore, kiwi berries in most areas of China from August to October reach commercial maturity. In the present study, kiwi berries were collected from Dandong (LD‐241, LD‐121), Benxi (HR), Taian (CJ‐1), and Tonghua (LD‐109, LD‐126, LD‐243, LD‐133) in China at the commercial maturity stage, and the sample collection time was showed in (Figure S1).

More than 80 fruits of nearly the same size without any disease or pest damage were randomly collected for each variety from four different regions. The samples were placed in cooler containers and immediately transported to the laboratory. Each kiwi berry variety was randomly divided into three groups (each group approximately 100–150 g) as three replicates for each experiment. Different kiwi berry varieties were washed and frozen at −20°C for no longer than 1 week, until phenolic extraction.

### 2.3 Phytochemical extraction of kiwi berry

Phenolics were extracted by previously proposed method with a slight modification (Gorinstein et al., 2009).

### 2.4 Total phenolic content assay

Total phenolic content was determined using the colorimetric Folin–Ciocalteu method according to Guo et al. (2012). The phenolic content was expressed as milligram gallic acid equivalents per 100 g in fresh weight (mg GAE/100 g FW). Data were reported as mean ± *SD* for three replications.

### 2.5 Total flavonoid content assay

Total flavonoid content was determined by the Sodium Borohydride/Chloranil‐Based (SBC) assay reported previously (He et al., 2008). Results were expressed as milligram catechin equivalents per 100 g fresh weight (mg CE/100 g FW). Data were reported as mean ± *SD* for three replications.

### 2.6 Vitamin C content assay

High‐performance liquid chromatography analysis was used to measure the vitamin C content. Vitamin C was extracted from 100 g kiwi berry with the mixture of 1% meta‐phosphoric and 1% perchloric acids. The chromatographic separation was performed on a Waters C18 column (4.6 mm × 250 mm, 5 μm). The mobile phase was consisted of A (formic acid:water = 0.1:99.9) and B (formic acid:acetonitrile = 0.1:99.9). Results were expressed as milligrams per 100 g fresh weight (mg/100 g FW). Data were reported as mean ± *SD* for three replications.

### 2.7 Qualitative analysis of phenolics by ultrahigh‐performance liquid chromatography coupled with quadrupole time‐of‐flight mass spectrometry and quantitative analysis of phenolics by high‐performance liquid chromatography coupled with photodiode array mass spectrometry

Ultrahigh‐performance liquid chromatography‐Q‐TOF‐MS was used for qualitative analysis of phenolics of eight kiwi berry varieties. Chromatographic separation was performed on the Dikma C18 column (3.0 mm × 150 mm, 5 μm). The mobile phase was consisted of A (formic acid:water = 0.1:99.9) and B (acetonitrile). The flow rate was 0.3 ml/min. Gradient elution system was as follows:

**Table:** 

0–1 min	1–3 min	3–12 min	12–17 min	17–22 min	22–30 min	30–55 min	55–58 min	58–65 min
10% B	10%–11% B	11%–15% B	15%–18% B	18%–20% B	20%–30% B	30%–40% B	40%–100% B	100%–10% B

Mass spectrometry was acquired by Q‐TOF‐MS (Bruker Compact+ H‐Class). The source parameters were as follows: negative iron mode; capillary voltage, 2.8 KV; dry gas (N_2_) flow, 3.0 L/min; temperature, 200°C. Inlet pressure of collision gas was 0.3 Bar for collision‐induced dissociation (CID). Collision energy was set to 10 eV. The mass spectrometry used software Bruker Compass Data Analysis 4.4.

High‐performance liquid chromatography‐PDA‐MS system (Shimadzu LCMS‐8050) was used for quantitative analysis of phenolics. The chromatographic column was the Dikma C18 column (4.6 mm × 250 mm, 5 μm). The mobile phase was consisted of A (formic acid:water = 0.1:99.9) and B (formic acid : acetonitrile = 0.1:99.9). Gradient elution system was as follows:

**Table:** 

0–1 min	1–3 min	3–5 min	5–17 min	17–22 min	22–28 min	28–33 min
10% B	10%–11% B	11%–12% B	12%–13% B	13%–14% B	14%–15% B	15%–17% B
33–38 min	38–48 min	48–53 min	53–54 min	54–63 min	63–68 min	68–80 min
17%–18% B	18%–20% B	20%–24% B	24%–25% B	25%–30% B	30%–100% B	100%–10% B

### 2.8 Peroxyl radical scavenging capacity assay

The antioxidant activities were determined by peroxyl radical scavenging capacity (PSC) assays as described previously (Adom & Liu, 2005). The vitamin C was utilized to make the standard curve with the concentration of 1, 2, 3, 4, and 5 μg/ml. Results were expressed as μmol vitamin C equivalents per 100 g in fresh weight (μmol VCE/100 g FW). Data were reported as mean ± *SD* for three replications.

### 2.9 Cellular antioxidant activity assay

The cellular antioxidant activity (CAA) assay was conducted as described previously (Wolfe & Liu, 2007). The CAA value was expressed as micromoles quercetin equivalents per 100 g fresh weight (μmol QE/100 g FW). Data were reported as mean ± *SD* for three replications.

### 2.10 Statistical analysis

Statistical analysis was performed using SPSS Statistics 17.0 (SPSS Inc.,). A one‐way analysis of variance was performed to determine the overall effect of different treatments, and Duncan's test was used for multiple comparisons; the significance level was set at *p* < .05. To establish a correlation between the phytochemical profile and antioxidant activities, bivariate Pearson's correlation analysis was performed using a two‐tailed test with IBM SPSS Statistics 17.0 for Windows (SPSS Inc.,) and multivariate correlation was conducted by partial least squares regression (PLS) using Unscrambler 10.1 (Camo Process AS). In the PLS method, the predictors (variable X) were the content of the phytochemical profile, with the responses (variable Y) being the PSC and CAA values.

## 3 RESULTS

### 3.1 Total phenolic content of eight kiwi berry varieties

**FIGURE 1:**
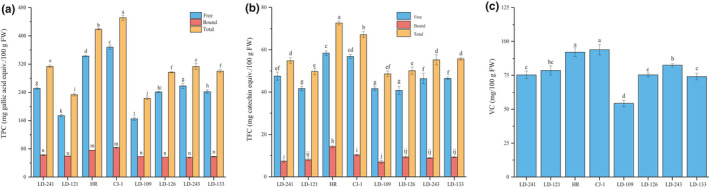
Total phenolic contents (TPCs) (a), total flavonoid contents (TFCs) (b), and vitamin C contents (c) of eight kiwi berry varieties (mean ± *SD*; *n* = 3). Bars with different letters differ significantly (*p* < .05)

The TPC of the eight kiwi berry varieties is presented in Figure 1a. The free TPCs ranged from 165.06 mg GAE/100 g FW (LD‐109) to 367.9 mg GAE/100 g FW (CJ‐1) and varied 2.23 times in the eight varieties. The free TPCs contributed 74.0% (LD‐109)–82.4% (LD‐243) of the total content, whereas the bound TPC was significantly lower than the corresponding free TPC, ranged from 55.21 mg GAE/100 g FW (LD‐243) to 83.26 mg GAE/100 g FW (CJ‐1). The total TPCs ranged from 223.09 mg GAE/100 g FW (LD‐109) to 451.16 mg GAE/100 g FW (CJ‐1).

### 3.2 Total flavonoid content of eight kiwi berry varieties

Total flavonoid contents of eight kiwi berry varieties are presented in Figure 1b. The decreasing order of free TFCs were LD‐126 > LD‐109 > LD‐121 > LD‐243 > LD‐133 > LD‐241 > CJ‐1 > HR. The free TFCs contributed 80.4% (HR)–86.8% (LD‐241) of the total content and significantly higher than corresponding bound TFCs.

### 3.3 Vitamin C content of eight kiwi berry varieties

Vitamin C contents of eight kiwi berry varieties are presented in Figure 1c. The highest vitamin C content was 93.94 mg/100 g FW found in CJ‐1. The average vitamin C content was 78.30 mg/100 g FW in these varieties.

### 3.4 Identification of phenolic compounds of eight kiwi berry varieties

The result of qualitative analysis of eight kiwi berry varieties is shown in Table 1. In the present study, three phenolic acids, three flavanols, and three flavonols were detected and identified by comparison with the retention time, UV spectra, MS spectra, MS/MS spectra of literature, and authentic standard: protocatechuic acid, caffeic acid, chlorogenic acid, proanthocyanidin B2, proanthocyanidin C1, (+)‐gallocatechin, quercetin‐3‐*O*‐galactoside, quercetin‐3‐*O*‐rutinoside, quercetin‐3‐*O*‐glucoside.

**TABLE 1:** Ultrahigh‐performance liquid chromatography coupled with quadrupole time‐of‐flight mass spectrometr qualitative analysis of the phenolic compounds of eight kiwi berry varieties

No	Retention time (min)	[M–H]^−^ (*m*/*z*)	MS/MS (*m*/*z*)	Phenolic compounds	Category
1	0.9	305.0982	248.0973	(+)‐gallocatechin^a^	Flavonoids
2	1.3	153.2474	109.7528	Protocatechuic acid^b^	Phenolic acid
3	1.4	577.1676	289.0871	Proanthocyanidin B2^a^	Flavanols
4	1.6	179.0502	135.0653	Caffeic acid^a^	Phenolic acid
5	2.2	353.1054	191.0667	Chlorogenic acid^b^	Phenolic acid
6	2.7	865.242	577.1562	Proanthocyanidin C1^a^	Flavanols
7	7.2	609.1838	300.0469	Quercetin‐3‐*O*‐rutinoside^a^	Flavonoids
8	7.3	463.1135	300.0411	Quercetin‐3‐*O*‐galactoside^a^	Flavonoids
9	7.9	463.1128	301.0539	Quercetin‐3‐*O*‐glucoside^a^	Flavonoids

^a^ Compared with the literature.

^b^ Compared with an authentic standard.

### 3.5 Phenolic composition of eight kiwi berry varieties

**FIGURE 2:**
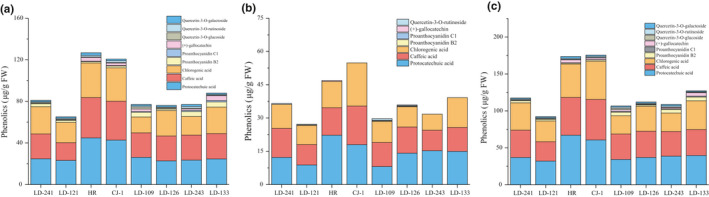
Free phenolic composition (a), bound phenolic composition (b), and total phenolic composition (c) of eight kiwi berry varieties (mean ± *SD*; *n* = 3)

The contents of phenolics of eight kiwi berry varieties are shown in Figure 2. Three phenolic acids, that is, protocatechuic acid, caffeic acid, and chlorogenic acid, were the predominant phenolics in kiwi berry. The average of protocatechuic acid, caffeic acid, and chlorogenic acid content was 29.07 μg/g FW, 26.63 μg/g FW, and 24.29 μg/g FW (free extracts); and 14.21 μg/g FW, 11.86 μg/g FW, and 11.26 μg/g FW (bound extracts), respectively.

Phenolic acids accounted 92.7% (protocatechuic acid 34.2%, caffeic acid 30.4%, chlorogenic acid 28.1%) of the total content of nine monomers. Phenolic acid is the main contributor of phenolic compounds in kiwi berry.

Three flavanols, that is, poanthocyanidin B2, proanthocyanidin C1, and (+)‐gallocatechin, were mainly found in kiwi berry free extracts. Bound flavanols were only found in small amounts in LD‐121 (0.26 μg/g FW poanthocyanidin B2), LD‐109 (0.24 μg/g FW poanthocyanidin B2, 0.23 μg/g FW poanthocyanidin C1), and LD‐126 (0.23 μg/g FW poanthocyanidin B2, 0.28 μg/g FW (+)‐gallocatechin). The average of total poanthocyanidin B2, proanthocyanidin C1, and (+)‐gallocatechin contents were 3.05 μg/g FW, 0.51 μg/g FW, and 2.04 μg/g FW, respectively.

Three flavonols, that is, quercetin‐3‐*O*‐galactoside, quercetin‐3‐*O*‐rutinoside, and quercetin‐3‐*O*‐glucoside, were found in small amounts in kiwi berry free extracts. The average of total quercetin‐3‐*O*‐galactoside, quercetin‐3‐*O*‐rutinoside, and quercetin‐3‐*O*‐glucoside contents were 0.85 μg/g FW, 1.35 μg/g FW, and 1.61 μg/g FW, respectively.

### 3.6 Rapid peroxyl radical scavenging capacity of eight kiwi berry varieties

**FIGURE 3:**
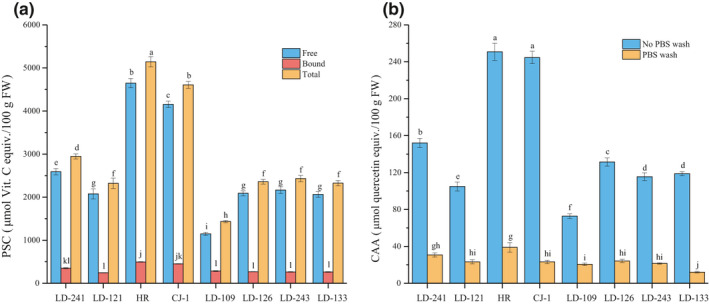
Peroxyl radical scavenging capacity (PSC) (a) and cellular antioxidant activity (CAA) (b) values of eight kiwi berry varieties (mean ± *SD*; *n* = 3). Bars with different letters differ significantly (*p* < .05)

Peroxyl radical scavenging capacity values of eight kiwi berry varieties are shown in Figure 3a. The free PSC values ranged from 1148.86 μmol VCE/100 g FW (LD‐109) to 4647.71 μmol VCE/100 g FW (HR), varied 4.05 times in eight varieties. The ratio of free PSC values to total PSC values ranged from 80.1% (LD‐109) to 90.4% (HR), whereas the bound PSC values ranged from 243.77 μmol VCE/100 g FW (LD‐121) to 495.96 μmol VCE/100 g FW (HR). The free PSC values were significantly higher than corresponding bound PSC values. The average of total PSC values was 2745.77 μmol VCE/100 g FW, ranged from 1433.54 μmol VCE/100 g FW (LD‐109) to 5143.67 μmol VCE/100 g FW (HR).

### 3.7 Cellular antioxidant activity of eight kiwi berry varieties

The CAA values of eight kiwi berry varieties are shown in Figure 3b. Since the CAA of the bound fraction was too low to be determined, only the CAA of the free fraction was determined. In no phosphate‐buffered saline (PBS) wash protocol, the average of CAA values was 148.85 μmol QE/100 g FW, ranged from 72.83 μmol QE/100 g FW (LD‐109) to 250.78 μmol QE/100 g FW (HR). In PBS wash protocol, the average of CAA values was 24.30 μmol QE/100 g FW.

### 3.8 Bivariate Pearson's correlation between phenolic compounds and antioxidant activities

**FIGURE 4:**
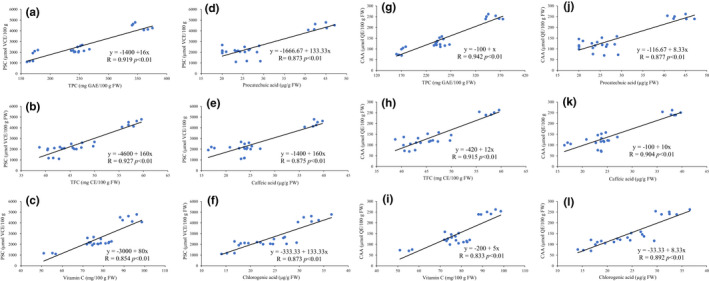
Bivariate correlation between phytochemical profile and antioxidant activities (peroxyl radical scavenging capacity [PSC], cellular antioxidant activity [CAA])

The linear relationship between different groups of phytochemical profile (TPC TFC phenolic acids) and antioxidant activities (PSC, no PBS wash‐CAA) of kiwi berry free extracts used Pearson's correlation was shown in Figure 4. PSC values showed strong correlation with TPCs (*R* = .919, *p* < .01) Figure 4a; TFCs (*R* = .927, *p* < .01) Figure 4b; Vitamin C contents (*R* = .854, *p* < .01) Figure 4c; protocatechuic acid contents (*R* = .873, *p* < .01) Figure 4d; caffeic acid contents (*R* = .875, *p* < .01) Figure 4e; and chlorogenic acid contents (*R* = 0.873, *p* < .01) Figure 4f. Significantly high correlation were also found between CAA (no PBS wash protocol) with TPCs (*R* = .942, *p* < .01) Figure 4g; TFCs (*R* = .915, *p* < .01) Figure 4h; Vitamin C contents (*R* = .833, *p* < .01) Figure 4i; protocatechuic acid contents (*R* = .877, *p* < .01) Figure 4j; caffeic acid contents (*R* = .904, *p* < .01) Figure 4k; and chlorogenic acid contents (*R* = .892, *p* < .01) Figure 4l.

Higher correlation coefficient values suggested a greater contribution by the phytochemical profile to the antioxidant activities of the kiwi berry free extracts. However, the contents of flavanols and flavonol monomers showed a weak correlation with antioxidative activities.

### 3.9 Multivariate correlation by PLS among phenolic compounds and antioxidant activities

**FIGURE 5:**
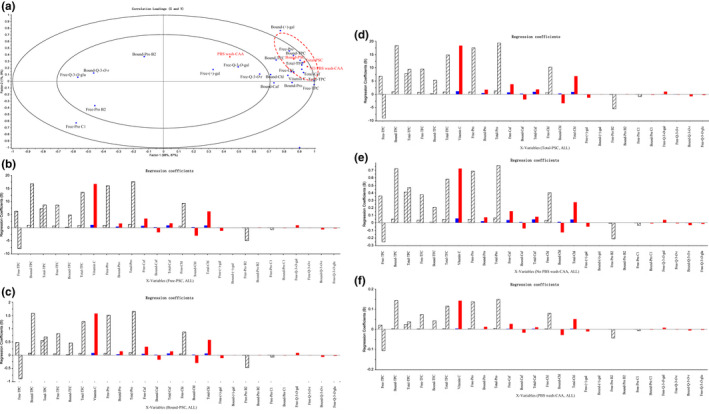
Partial least squares regression (PLS) plots of the correlations between phytochemical profile and antioxidative activities in kiwi berry extracts (a). Regression coefficients of different phytochemical profile on antioxidative activity (free peroxyl radical scavenging capacity [PSC] (b), bound PSC (c), total PSC (d), no PBS wash‐cellular antioxidant activity (CAA) (e), PBS wash‐CAA (f)). The antioxidative activities are in red font, and phytochemical profile are in blue font. Abbreviations of phenolic compounds: (+)‐gal, (+)‐gallocatechin; Caf, Caffeic acid; Chl, Chlorogenic acid; Pro B2, Poanthocyanidin B2; Pro C1, Poanthocyanidin C1; Pro, Protocatechuic acid; Q‐3‐*O*‐gal, Quercetin‐3‐*O*‐galactoside; Q‐3‐*O*‐glu quercetin‐3‐*O*‐glucoside; Q‐3‐*O*‐r quercetin‐3‐*O*‐rutinoside

The multivariate correlation used the PLS regression models of various groups of the phytochemical profile (phenolics, flavonoids, and vitamin C as well as individual phenolic compounds) with the antioxidant activity (PSC and CAA) of the eight kiwi berry varieties shown in Figure 5. The PLS plots of the kiwi berry extracts where 98.59% of the phytochemical profile explained 95.77% of the variation in the antioxidant activities in two factors.

As shown in Figure 5, TPCs, vitamin C, and protocatechuic acid contents positively correlated with free PSC (B), bound PSC (C), and total PSC (D) values. Some moderate contributions were also found in TFCs and chlorogenic acid contents. As shown in Figure 5e, TPCs, TFCs, and vitamin C contents were positively associated with CAA (no PBS wash protocol) values. However, as shown in Figure 5f, the contents of the phytochemical profile did not show a close correlation with CAA (PBS wash protocol) values. These were in agreement with the results of the bivariate correlation analysis.

## 4 DISCUSSION

### 4.1 Phytochemical profile of eight kiwi berry varieties

Consumption of fruits and vegetables is known to protect against chronic conditions because they are an excellent source of biologically active compounds, including phenolics, flavonoids, and vitamin C (Aghdam et al., 2020; del Río‐Celestino & Font, 2020). The kiwi berry (*A*. *arguta*) is close to the top of fruits classified as “health food” or “superfood,” because it contains more than 20 essential nutrients, and a series of vitamins (Wang et al., 2018). It is considered the richest sources of vitamin C with up to 430 mg/100 g fresh weight (FW) and phenolics with up to 389.7 mg GAE/100 g FW, and flavonoids containing up to 130.4 mg CE/100 g FW in commonly consumed kiwi berry (del Río‐Celestino & Font, 

Phenolics are strong antioxidants and can be found in many fruits and vegetables (Li et al., 2019; Liu, 2013). Many reports have indicated that the phenolics in most fruits are in a free form (Sun et al., 2002). Free phenolics are rapidly released and absorbed in the stomach and small intestine (Jing et al., 2008); they reduce the oxidative stress response of cells and prevent chronic diseases (Jing et al., 2008). The bound phenolics in the kiwi berry are retained in the stomach and intestines after digestion and are released in the colon during fermentation by colonic bacteria, thus preventing gastrointestinal cancers, breast cancers, and prostate cancers (Adom & Liu, 2002).

Similar to phenolics, flavonoids exist in both free and bound form, and the role of bound flavonoids cannot be ignored (Romagnolo & Selmin, 2012). It has been reported that bound flavonoids can be absorbed by the intestinal membrane and then partially converted to glucuronic acid and sulfate (Liu, 2007).

It was reported that the TPC of kiwifruit (*A*. *deliciosa*) was 42.34 mg GAE/100 g FW (Saeed et al., 2019), which is lower than kiwi berry (*A*. *arguta*) (318.76 mg GAE/100 g FW) in the present study. Similarly, the TFC of kiwifruit was previously reported as 36.30 mg CE/100 g FW (Saeed et al., 2019), which is lower than that of the kiwi berry (56.75 mg CE/100 g FW) in the present study. Therefore, the kiwi berry is a richer source of various phytochemicals than kiwifruit.

In this study, the vitamin C content of kiwi berries ranged from 54.32 to 93.94 mg/100 g FW, which was higher than that of orange (51 mg/100 g FW), strawberry (29–48 mg/100 g FW) (Iqbal et al., 2004), and traditional kiwifruit (Ewa Baranowska‐Wójcik, 2019). The results of these studies showed that kiwi berry was a good vitamin C dietary supplement. Moreover, kiwifruit had a lot of hair on the surface of the peel, rendering it unsuitable for consumption. In contrast, the kiwi berry skin is mostly smooth and without hair, which makes the whole fruit more suitable for direct consumption without removing the skin (Park et al., 2014). Therefore, the kiwi berry is a richer source of various phytochemicals than kiwifruit.

### 4.2 Phenolic composition of eight kiwi berry varieties

In the present study, phenolic acids were the most common antioxidant phytochemicals in kiwi berry, with a significant amount of protocatechuic acid (CJ‐1 50.68 μg/g FW 43.7%), caffeic acid (LD‐109 44.74 μg/g FW 35.1%), and chlorogenic acid (LD‐133 47.67 μg/g FW 31.7%). The contents of protocatechuic acid (0.243–1.394 μg/g FW) and caffeic acid (0.002–0.331 μg/g FW) in kiwifruit (Saeed et al., 2019) were lower than those of kiwi berry (*A*. *arguta*) (22.77–33.71 μg/g FW and 23.54–24.33 μg/g FW, respectively) in the present study. Similarly, the contents of chlorogenic acid (0.007–0.07 μg/g FW) and caffeic acid (0.009–0.04 μg/g FW) in kiwifruit (Latocha et al., 2010) were lower than those in kiwi berry (17.85–26.19 μg/g FW and 23.54–24.33 μg/g FW, respectively) in the present study.

Protocatechuic acid has a variety of biological activities, including antibacterial, antioxidant, anti‐inflammatory, antihyperglycemic, and neuroprotective effects (Tseng et al., 2000). In addition, protocatechuic acid has the potential for chemical protection, which inhibits chemical carcinogens in vitro and produces proapoptotic and antiproliferative effects in different aspects (Tseng et al., 2000). Caffeic acid and chlorogenic acid have effective 2,2′‐azinobis(3‐ethylbenzothiazoline‐6‐sulfonic acid) (ABTS^•+^), 2,2‐diphenyl‐1‐picryl‐hydrazyl‐hydrate (DPPH^•^), and superoxide anion radical scavenging activities (Gulcin, 2006). These results also indicate that kiwi berry is a good source of phenolic acids.

### 4.3 Antioxidant activities of eight kiwi berry varieties

Oxidative stress is involved in the pathology of human aging and a variety of chronic diseases (Liu, 2007). Dietary antioxidants can fight oxidative stress in the body and maintain a balance between oxidants and antioxidants, thereby protecting the cells from reactive oxygen species (ROS) and free radicals. Fruits rich in natural antioxidants could reduce the risk of oxidative stress‐related diseases (Liu, 2007). It has been reported that kiwi berry is an important source of bioactive compounds and significantly contributes to human health (Latocha et al., 2015). The free PSC values of kiwi berry (5143.67 μmol VCE/100 g FW) were significantly higher than those of kiwifruit (55.58 μmol VCE/100 g FW) (Saeed et al., 2019). Moreover, kiwi berry had higher free PSC values than apple (309.2 μmol VCE/100 g FW), cranberry (1019.9 μmol VCE/100 g FW), and grape (2018.9 μmol VCE/100 g FW) (Adom & Liu, 2005). The results show that the free fractions of kiwi berry have a potent ability to scavenge free radicals and could prevent chronic diseases. In this study, the bound PSC values were lower than the corresponding free PSC values, mainly due to the higher TPC and vitamin C content in the free fractions than in the bound fractions. The bound antioxidant substance can survive digestion in the stomach and intestines and be released in the colon by microbial fermentation, which would help prevent digestive system diseases and breast and prostate cancers (Adom & Liu, 2002). In the no PBS wash protocol, the CAA values of kiwi berry (72.83–250.78 μmol QE/100 g FW) were higher than those of kiwifruit (55.68–121.72 μmol QE/100 g FW) (Saeed et al., 2019). Similarly, in the PBS wash protocol, the CAA values of kiwi berry (12.05–38.92 μmol QE/100 g FW) were significantly higher than those of kiwifruit (2.32–5.85 μmol QE/100 g FW) (Saeed et al., 2019). The results suggested that kiwi berry was a better source of antioxidants than kiwifruit, at the cellular level.

Antioxidant assays have become an important tool for selecting antioxidants that are beneficial to human health; therefore, it is important to choose a favorable antioxidant method to determine the antioxidant activity of fruits, vegetables, and dietary supplements (Sun et al., 2002). In this study, the total antioxidant activities of the kiwi berries were measured using the PSC and CAA assays. The PSC method was used in the in vitro antioxidant activity assay, which has several advantages over the previous DPPH, ABTS (Park et al., 2011), and ferric reducing antioxidant power (FRAP) (Latocha et al., 2015) methods. Unlike the chemically synthesized DPPH and ABTS radicals, the peroxyl radicals used in the PSC assay are naturally present in the human body. In addition, the FRAP assay is conducted at pH 3.6, which is not the normal condition of the human body. Nevertheless, the PSC assay was performed in a neutral environment (pH = 7.4), similar to that of the human body. The CAA method was used in the in vivo antioxidant activity assay (Wolfe & Liu, 2007), which was used to determine the intracellular antioxidant activity of kiwi berry. In the CAA assay, antioxidants from the extracts could be absorbed into the cells, could react with the ROS intracellularly, or break the peroxyl radical chain reactions at the cell surface by interacting with the cell membrane (Wolfe et al., 2008). The CAA values may lead to a reasonable correlation with the in vivo antioxidant activity. The PBS wash protocol evaluated the CAA of the phytochemicals that could easily pass through cells.

### 4.4 Correlation among phytochemical profile and antioxidant activities

Previous studies have illustrated that both intrinsic and extrinsic factors such as genetic, environmental, maturity, postharvest handling, and storage lead to differences in the phenolic composition among the fruits (Latocha et al., 2015). The results of this study provide important information on the phytochemical profile and antioxidant activities of eight kiwi berries from four regions in China (Figure S1). The results of bivariate Pearson's analysis suggest the significant regression coefficient of TPCs, TFCs, and vitamin C to the total antioxidant activities (PSC, no PBS wash‐CAA). The proportion of three phenolic acids (protocatechuic acid, caffeic acid, and chlorogenic acid) to TPC was 92.1%. This indicates that phenolic acids contributed more to the antioxidant activities than other phenolic monomers. In summary, the contribution of antioxidant activities of kiwi berry extracts mainly depends on the phenolics, flavonoids, and vitamin C. The results of multivariate correlation analysis indicate that TPCs (phenolic acids), TFCs, and vitamin C have the greatest impact on antioxidant activities. In view of this, HR (located in Benxi, LN) and CJ‐1 (located in Taian, SD) were selected as the better varieties. The results provide a theoretical basis for the selection of kiwi berry varieties and the development and utilization of functional foods.

## 5 CONCLUSION

Conclusively, as the variety of kiwi berry continues to increase, people's interest in identification, both in terms of quantity and quality, also has been increased. In addition, the number of published studies on the nutritional value and health benefits of kiwi berry has been a significant increase. This study aimed to assess and summarize the phytochemical profile and antioxidant activities of eight common kiwi berry varieties in China, exploring their potential functional properties in foodstuffs. The results demonstrate the differences in phytochemical profiles and antioxidant activities among different kiwi berry varieties, which will help to form useful guidance for plant breeders, food scientists, and consumers to provide a theoretical basis for the selection of kiwi berry varieties and the development and utilization of functional foods.

Kiwi berry's medicinal nutrients have prompted continuous research on its antioxidant, antitumor, and anti‐inflammatory properties. Other health‐related properties of kiwi berry, including immunological benefits, have not been fully studied or described, but given the growing popularity of this fruit, such research is undoubtedly necessary.

## CONFLICT OF INTEREST

The authors declare no competing financial interest.

## Supporting information

Fig S1‐S2

## Data Availability

The original contributions presented in the study are included in the article/Supplementary Material, further inquiries can be directed to the corresponding authors.
