# Supplementary material for: Assessment of the phytochemical profile and antioxidant activities of eight kiwi berry (Actinidia arguta (Siebold & Zuccarini) Miquel) varieties in China
Source: Food Sci Nutr. 2021 Aug 20;9(10):5616–25. doi: 10.1002/fsn3.2525 (PMC8497840; doi:10.1002/fsn3.2525)
Supplement: Supplementary file 1 — Fig S1‐S2 [file FSN3-9-5616-s001.docx]

| Varieties | Collection time | Origin | Location |
| --- | --- | --- | --- |
| LD-241 | 2018.09.28 | Dandong, LN | 124°23^’^E, 40°07^’^N |
| LD-141 | 2018.09.28 | Dandong, LN | 124°23^’^E, 40°07^’^N |
| Huairou (HR) | 2018.08.29 | Benxi, LN | 124°17^’^E, 41°24^’^N |
| Changjiangyihao (CJ-1) | 2018.09.25 | Taian, SD | 117°06^’^E, 36°11^’^N |
| LD-109 | 2018.10.02 | Tonghua, JL | 125°93^’^E, 41°73^’^N |
| LD-126 | 2018.10.02 | Tonghua, JL | 125°93^’^E, 41°73^’^N |
| LD-243 | 2018.10.02 | Tonghua, JL | 125°93^’^E, 41°73^’^N |
| LD-133 | 2018.10.02 | Tonghua, JL | 125°93^’^E, 41°73^’^N |

**Supplementary Figure 1.** The sample collection date and locations of eight kiwi berry varieties.


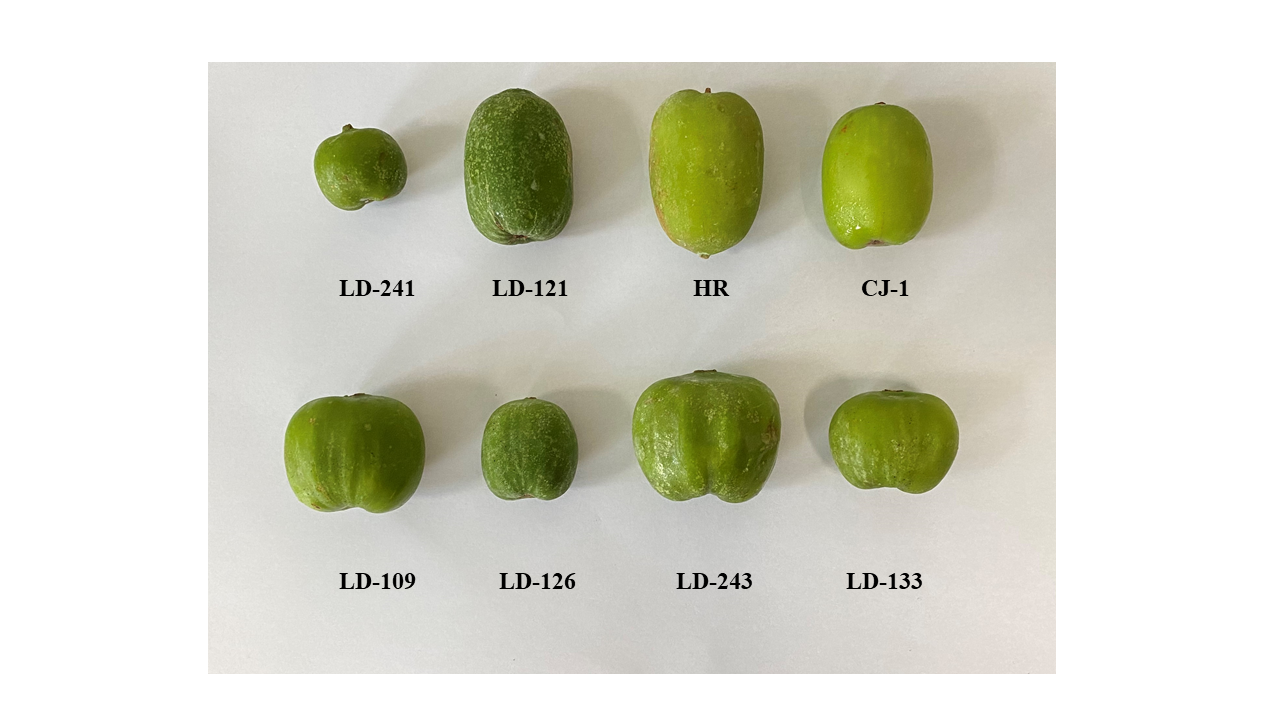


**Supplementary Figure 2.** The photographs of eight kiwi berry varieties.
